# New method for treatment of inferior vena cava tumor thrombus – case study

**DOI:** 10.3325/cmj.2015.56.139

**Published:** 2015-04

**Authors:** Zoltán Nagy, Endre Gyurkovics, Péter Pajor, Mária Tarjányi, Attila Szijártó, Sandor G. Vari

**Affiliations:** 1 Department of Surgery, Bajcsy-Zsilinszky Hospital, Budapest, Hungary; 2 1^st.^ Department of Surgery, Semmelweis University, Budapest, Hungary; 3 International Research and Innovation Management Program, Cedars-Sinai Medical Center, Los Angeles, CA, USA

## Abstract

Conventional surgical therapy for advanced renal venous tumor thrombi results in high morbidity, so there is a need for less invasive techniques. This report presents the first case of a successful inferior vena cava (IVC) tumor thrombus removal without complications with balloon catheter (BC) via internal jugular vein (IJV), called the venous tumor thrombus pushing with balloon catheter (VTTP BC). Under the control of transesophageal echocardiogram and fluoroscope, a balloon catheter was sleeved on the guide wire, which was already inserted into the right internal jugular vein (IJV) and was driven distally above the IVC tumor thrombus. The balloon was inflated to occlude the IVC for prevention of pulmonary embolization. After the occlusion, the guide wire was driven to the cavotomy and was opened at the ostium of the right renal vein. It was pulled at both ends and stretched to serve as a rail. The balloon was gently pushed toward the cavotomy and the thrombectomy was completed. This is a less invasive method for treatment of venous tumor thrombus level 3 that can reduce surgical time, blood loss, and complication rates compared to the existing surgical methods. Also, it can be performed without thoracotomy, cardiopulmonary bypass, hypothermic circulatory arrest, and liver mobilization.

Venous tumor thrombus (VTT) develops in 4 to 15% of renal cell carcinomas (RCCs) and usually propagates into the renal vein and the inferior vena cava (IVC); in 1% of the cases it even propagates into the right atrium (1). Therapy for advanced cases complicated with VTT requires a multidisciplinary approach including a urologist, general, vascular, and cardiac surgeon, anesthesiologist, and oncologist. The basis of such therapy is a radical nephrectomy and VTT resection. Neves and Zincke (2) defined four stages of VTT propagation to facilitate the prognosis and therapeutic decision-making: Level 1 – renal vein thrombus, 2 – infrahepatic IVC, 3 – intrahepatic IVC, but below the hepatic veins, and 4 – above level 3. Level 4 thrombi can be suprahepatic, supradiaphragmatic, or intraatrial. The standard surgical technique for the management of level 3 and 4 thrombi is thoracoabdominal tumor resection with cardiopulmonary bypass (CPB) (3). Due to the high morbidity resulting from complications associated with these approaches, new and less invasive techniques have been developed (4,5). It was found that in patients who had undergone inferior vena cava thrombectomy with radical nephrectomy, the level of tumor thrombus was not an independent prognostic factor (6), but the important prognostic factors were pathological stage, nuclear grade, tumor histology, lymph node and distant metastatic status, preoperative performance status, Charlson comorbity index, and nutritional status of the patient (7). This report presents the first case of a successful IVC tumor thrombus removal without complications with balloon catheter (BC) via internal jugular vein (IJV).

## The patient and methods

A 58-year-old male patient had a history of hypertension and type 1 diabetes. In 2009, he was diagnosed with kidney tumor on both sides and underwent right nephrectomy. The histopathology result was clear cell renal cell cancer (RCC), T3N0MX. In April 2010, a left partial resection of the kidney was performed. Following both surgical interventions, the patient received chemotherapy as an adjuvant oncological treatment with Vinblastin and Roferon A. The VTT was diagnosed in October 2010, the oncology treatment regime was changed preoperatively, and Stutent was administrated.

**Figure 1:**
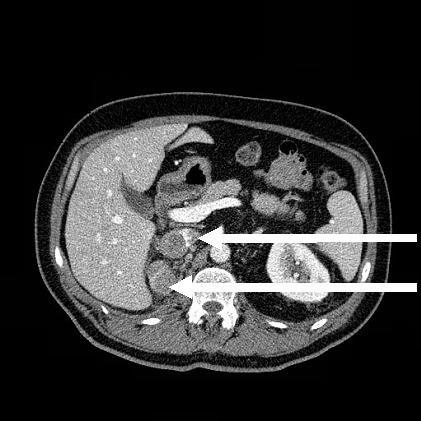
Preoperative CT scan: upper arrow pointing to the inferior vena cava with tumor thrombus, the arrow bellow pointing to the metastasis.

Half a year after the second operation, abdominal ultrasound and preoperative thoracoabdominal CT scans showed a 5 cm-diameter metastasis in the right adrenal gland, an IVC tumor thrombus, a left hepatic vein tumor thrombus, and four 3 mm large metastases in the lungs (Figure 1). MRI and transesophageal endoscopy (TEE) were used for the assessment of the proximal extension of the VTT. A level 3 tumor thrombus expanding to the hepatic veins was detected. The patient’s general condition was good, but the renal function gradually deteriorated 2 weeks before surgery. Preoperative se-creatinine was 230 µmol/L and blood urea nitrogen (BUN) was 15.1 mmol/L with glomerular filtration rate (GFR) 25.5 mL/min/L.73 m^2^, sodium 141 mmoL/L, and potassium 5.6 mmol/L.

The goal of the surgery was to normalize the venous flow of the left kidney, improve renal function, and reduce tumor volume with the removal of both the adrenal gland metastases and the VTT, which would improve the effectiveness of the oncological treatment. The surgery was done under intratracheal narcosis with an antibiotic prophylaxis (2 g ceftriaxone, 500 mg metronidazole). The intervention was guided by TEE and fluoroscope. The patient was placed in a supine position with additional elevation of the liver area. The operation was performed through partial Chevron incision. A 5 cm tumor was found in the right adrenal gland, attached to the liver-IVC angle. After the mobilization of the duodenum, the infiltrated conglomerate of the lymph nodes was dissected from the anterior and right surface of the IVC, and the tumorous adrenal gland was removed *en bloc*. The IVC section was looped with a rubber band above and below the tumorous infiltrated right renal vein stump. The same method was used for the left renal vein, which was significantly dilated but the lumen was tumor-free. The IVC VTT blanketed the left renal vein opening and did not proliferate into the vein. However, the tumor mass reduced the renal vein flow, increased the intravenous pressure, and dilated the vein.

**Figure 2:**
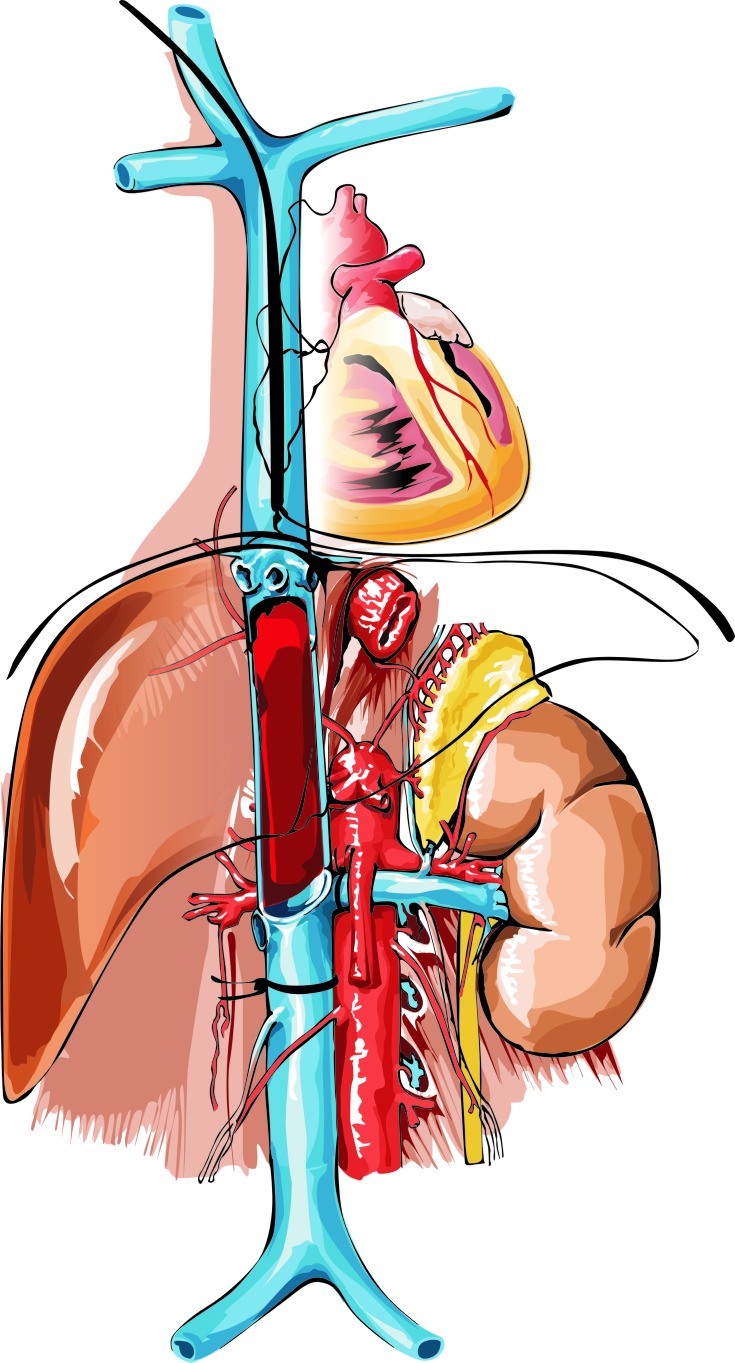
A guide wire was placed through the right internal jugular vein to the level of the diaphragm.

**Figure 3:**
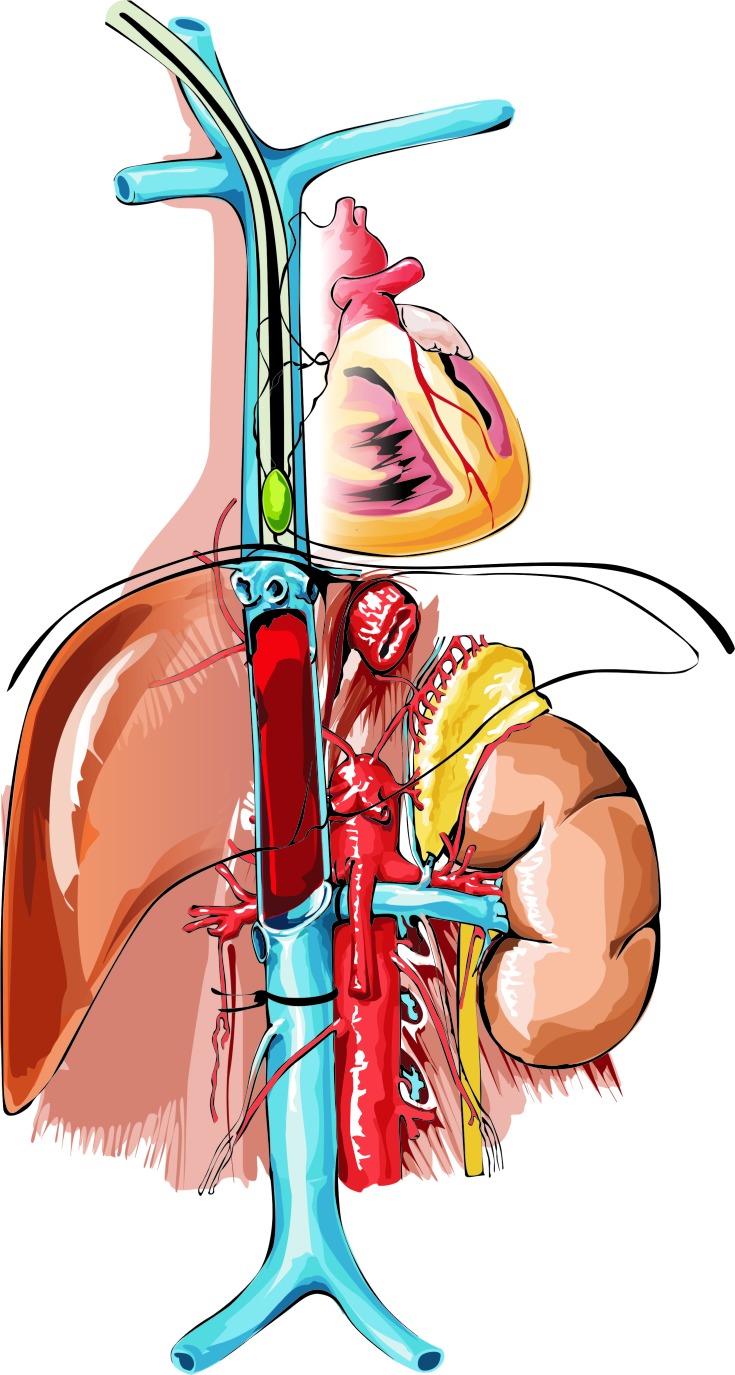
A balloon catheter was inserted with the help of the guide wire to the level of the diaphragm.

After the ultrasound-guided puncture of the right IJV, under fluoroscopic control a guide wire was introduced and steered to the level of the diaphragm (Figure 2). A catheter (size (F) 8.5; sheath sizing (F) 16; shaft length 100 cm; balloon diameters 30 mm; balloon lengths 6 cm with 2 cm narrowed end) (Large Omega NV Valvuloplasty Balloon Catheter, Cook Medical Europe Ltd, Limerick, Ireland) was guided until it reached the diaphragm, where the balloon was inflated (Figure 3). Before the balloon was dilated, 10.000 IU heparin sodium was administered for systemic heparinization.

**Figure 4:**
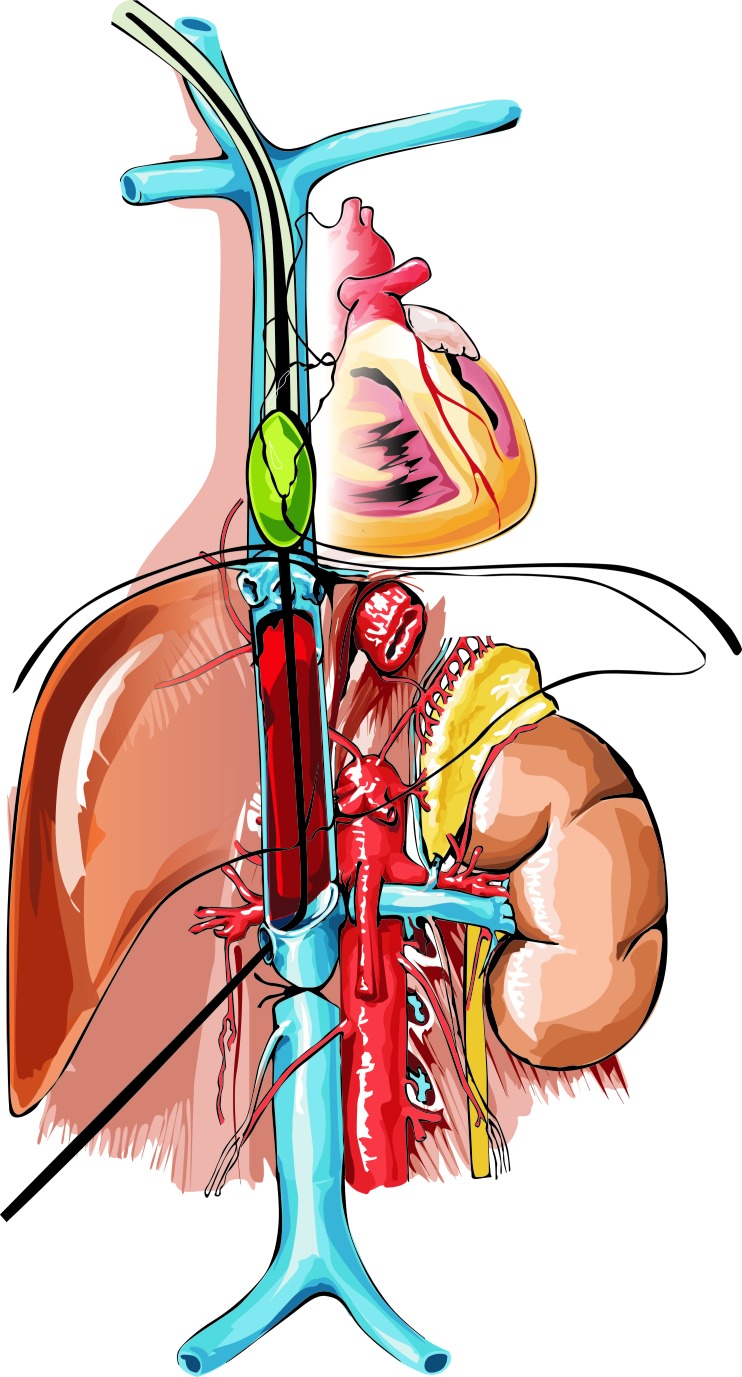
Following balloon inflation the guide wire was pushed further to the cavotomy and then the end of the wire was pulled out of the vein.

The balloon size was determined by the cava diameter measured preoperatively. The balloon was inflated at the level of the diaphragm to obstruct the IVC. In the next step, the left renal vein, and consecutively the IVC bellow left renal vein, were occluded using rubber loops for clamping. Further bleeding from the hepatic veins was prevented with the Pringle maneuver. Venotomy was performed with an excision of the tumorous right renal vein ostium. Following balloon inflation, the guide wire was pushed further to the cavotomy and the end of the wire was pulled out of the vein (Figure 4). A part of the thrombus was removed through the cavotomy.

**Figure 5:**
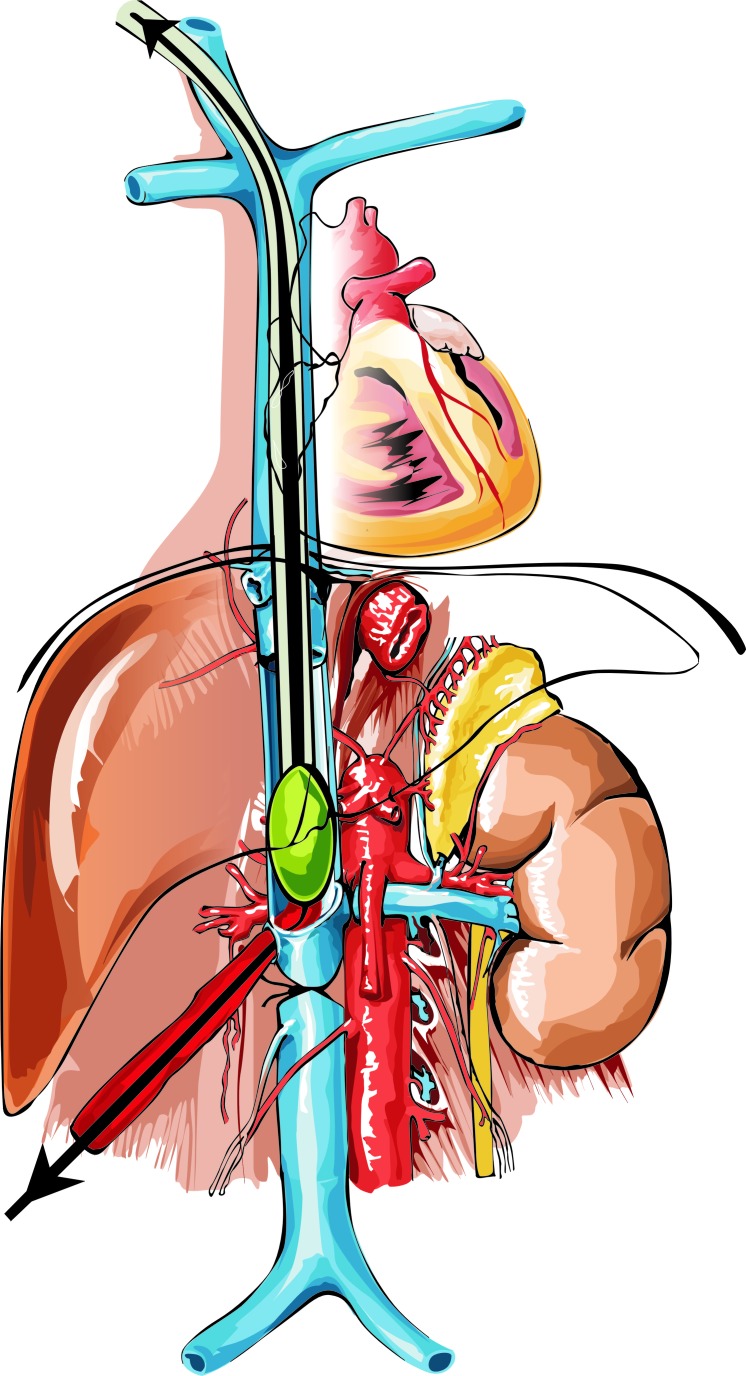
During thrombectomy the wire stretched to serve as rail, and was used as a trajectory to deliver pressure and gently push the tumor thrombus toward cavotomy, which resulted in successful removal.

During thrombectomy, the wire stretched to serve as rail. It was used as a trajectory to deliver pressure on the tumor thrombus and was slowly pushed toward to the cavotomy, which resulted in successful tumor thrombus removal (Figure 5). After the partial retraction of the guide wire, the catheter was cut proximally to the inflated balloon and pulled out through the IJV. The preoperative CT images showed the involvement of the left hepatic vein, so after the completion of the Pringle maneuver there was a chance for embolus dissemination. For this reason, a protective cava-filter (Braun Aesculap cavafilter, B. Braun Melsungen AG, Melsungen & Aesculap AG, Tuttlingen, Germany) was inserted through the guide wire to avoid embolization. The filter was positioned at the level of the diaphragm above the opening of hepatic veins. In general, it is not necessary to use cava filter during VTT thrombectomy.

After positioning the filter, the guide wire was pulled out proximally. Then the cava was clamped above the cavotomy with a rubber loop and the Pringle maneuver was loosened up. When the opened cava was explored, no tumor remains were found. After degassing, the cavotomy was closed with running stitches. The circulation recovered after loosening up the caval exclusion and no signs of embolization were observed. After the suspension of heparinization, drainage and layered closure with continuous suture were done. The surgery lasted 4 hours and 20 minutes. The patient received 4 × 200 mL red blood cell concentrate and 3 × 200 mL fresh frozen plasma and arrived intubated at the intensive care unit. After the surgery, the patient’s urine output was 3100 mL. Heparin sodium 750 IU/h was administered with an infusion pump for anticoagulation. On the first postoperative day, the dose was increased to 1000 IU/h, the patient was extubated, and the empty cava filter was removed. On the third day, mobilization started; on the fourth day low-molecular-weight heparin (LMWH) was initiated; and on the fifth day the intestinal passage began. The patient was released on the eighth day. The wound healing was *per primam* and the stitches were removed on the 14th day. There were no postoperative complications. Renal function returned to normal and the patient survived one year.

## Discussion

This study shows that our newly developed VTTP BC method can be applied in cases of level 3 thrombi. The IVC can be securely occluded during the operation-with the balloon catheter safely placed above the IVC tumor thrombus under fluoroscopic and TEE monitoring, thus avoiding the danger of embolization. A further advantage of the VTTP BC method is the less invasive resection of thrombi expanding above the level of the hepatic veins. After thrombectomy, the IVC can be clamped bellow the liver and the IVC resection and reconstruction can be done if necessary.

The basic therapy for RCC complicated with VTT is radical nephrectomy combined with tumor thrombectomy, though the resection and reconstruction of the IVC and metastasectomy may be necessary. The incidence of perioperative complications depends on the cephalic expansion of the VTT. Level 1 and 2 thrombi are easily resectable, with low complication rates by laparotomy alone. Level 3 or 4 thrombi can be resected with extensive hepatic mobilization or the use of CPB. The introduction of CPB (8) and hypothermic circulatory arrest (HCA) (9) made dissection of such tumors possible and decreased the incidence of embolization. However, CPB prolongs the length of the operation and may result in significant blood loss, ischemic lesions in the central nervous system and other organs, and postoperative coagulopathy (10). The perioperative complication rate in cases combining CPB with HCA can reach 31% (1).

Some research groups have successfully applied endoluminal IVC occlusion to reduce morbidity. Zini et al (11) applied cavotomy below the level of the thrombus and resected level 2 and 3 thrombi under continuous TEE monitoring, occluding the IVC with a balloon catheter pushed above the tumor thrombus and pulling the thrombus out of IVC. The application of this procedure successfully reduced the rate of major complications in VTT resections, including embolization. However, Zini’s technique still entails a considerable risk of embolism since the catheter is guided to the thrombus (12).

Rigberg et al (13) performed suprahepatic IVC exclusion via a balloon catheter inserted through the IJV. This technique, however, is limited to the treatment of level 2 thrombi, and positioning the balloon for proper exclusion is difficult. A further problem is the disturbance of hepatic venous effluence by the suprahepatic occlusion, a complication which is challenging for the patient. Also, Pringle’s maneuver is not performed. Yang et al (14) applied a less invasive method, similar to Rigberg’s et al (13), on 10 patients with level 2 VTT. In this technique, surgeon occludes the suprahepatic, infraphrenic IVC with a balloon placed through the IJV. IVC occlusion was tolerable in 90% of the cases, and no major postoperative complications developed. Eight patients were alive and tumor-free at the last follow-up. Metcalfe et al (15) modified Yang’s technique by holding the mean arterial pressure at a constant value and using an intraoperative cavogram. Kanka et al (16) slightly modified these methods for the resection of a level 3 VTT. They applied IVC exclusion above the level of the thrombus with an inflatable balloon inserted through the right IJV. This, however, required a sufficient distance between the VTT and the ostium of the hepatic veins and could not be applicable in cases of long thrombi.

Before VTTP BC method was developed in 2011, in 10 years the authors had performed surgeries in 33 patients to remove RCC combined with tumor thrombus in the IVC. In these patients, all deaths were caused by the postoperative progression of the underlying disease. Nevertheless the ten years’ experience with level 3 caval vein tumor thrombus indicated that it can be removed by a less aggressive surgical approach (17).

A good candidate for safe thrombectomy procedures is the AngioVac (AngioDynamics, Latham, NY, USA) procedure, which has already been used for percutaneous removal of right atrial and vena caval thrombi, vegetation, and tumors. This procedure provides an alternative to open surgical resection for high risk patients who are regularly poor surgical candidates and unsuitable for thrombolysis. Although AngioVac can reduce the risk of embolization, it was not found to be a better method to reduce the metastasis during the removal of tumor thrombi (18,19).

VTTP BC method developed by our team is similar to Zini’s method (11), since both techniques use balloon catheters. The difference is that in VTTP BC the balloon catheter is inserted into the IJV and used to push out the tumor thrombus from the proximal to distal position. In the Zini’s method, it is inserted through the cavotomy and pushed proximally above the tumor thrombus.

A major disadvantage of VTTP BC is the difficulty of pushing the balloon distally even with strained guide wire. This is why the vector of the force in short length in Zini’s method provides a better movement control than the vector of the force on the long axis of the wire in VTTP BC method. The use of the catheter without a guide wire is very risky since it could perforate the vessel during the forced driving of the tumor thrombus distally in IVC. Development of a proper junction between the end of the balloon inserted into IJV, positioned above the tumor thrombus, inflated, and a pull-off stalk placed through the cavotomy could lead to better results. With such a modification, this method could also be used with Fogarty’s embolectomy.

In conclusion, this report shows that the less invasive VTTP BC method offers significant clinical benefits since it does not require the use of thoracotomy, cardiopulmonary bypass, hypothermic circulatory arrest, and liver mobilization, and can better control the risk of embolization.
